# Impact of Nosema Disease and American Foulbrood on Gut Bacterial Communities of Honeybees *Apis mellifera*

**DOI:** 10.3390/insects12060525

**Published:** 2021-06-06

**Authors:** Poonnawat Panjad, Rujipas Yongsawas, Chainarong Sinpoo, Chonthicha Pakwan, Phakamas Subta, Sasiprapa Krongdang, Ammarin In-on, Siriwadee Chomdej, Panuwan Chantawannakul, Terd Disayathanoowat

**Affiliations:** 1 Department of Biology, Faculty of Science, Chiang Mai University, Chiang Mai 50200, Thailand; poonnawat_p@cmu.ac.th (P.P.); rujipas_y@cmu.ac.th (R.Y.); chainarong_s@cmu.ac.th (C.S.); chonthicha_pa@cmu.ac.th (C.P.); phakamas_subta@cmu.ac.th (P.S.); siriwadee.ch@cmu.ac.th (S.C.); panuwan.c@cmu.ac.th (P.C.); 2 Faculty of Science and Social Sciences, Burapha University Sakaeo Campus, Sakaeo 27160, Thailand; sasiprapa.kr@buu.ac.th; 3 Bioinformatics & Systems Biology Program, King Mongkut’s University of Technology Thonburi (Bang Khun Thian Campus), Bang Khun Thian, Bangkok 10150, Thailand; ammarin.ammarinin@mail.kmutt.ac.th; 4 Center of Excellence in Bioresources for Agriculture, Industry and Medicine, Chiang Mai University, Chiang Mai 50200, Thailand; 5 Center of Excellence in Microbial Diversity and Sustainable Utilization, Chiang Mai University, Chiang Mai 50200, Thailand

**Keywords:** *Apis mellifera*, *Nosema ceranae*, American Foulbrood, 454-pyrosequencing

## Abstract

**Simple Summary:**

*Nosema ceranae* and *Paenibacillus larvae* are virulent pathogenic microbes in honeybee adults and larvae, respectively. However, there is still a lack of data on their dysbiosis effect on gut bacteria in honeybees. The guts of control and infected bees of both diseases were analyzed for bacterial communities by next-generation sequencing. The statistical data showed that *Nosema ceranae* affected bacterial dysbiosis in the adult honeybees’ guts. The guts of *Paenibacillus larvae*-infected honeybee larvae did not differ from the guts of larvae not infected with *P. larvae*. These data could be applied to control pathogens in the apicultural industry.

**Abstract:**

Honeybees, *Apis mellifera*, are important pollinators of many economically important crops. However, one of the reasons for their decline is pathogenic infection. Nosema disease and American foulbrood (AFB) disease are the most common bee pathogens that propagate in the gut of honeybees. This study investigated the impact of gut-propagating pathogens, including *Nosema ceranae* and *Paenibacillus larvae,* on bacterial communities in the gut of *A. mellifera* using 454-pyrosequencing. Pyrosequencing results showed that *N. ceranae* was implicated in the elimination of *Serratia* and the dramatic increase in *Snodgrassella* and *Bartonella* in adult bees’ guts, while bacterial communities of *P. larvae-*infected larvae were not affected by the infection. The results indicated that only *N. ceranae* had an impact on some core bacteria in the gut of *A. mellifera* through increasing core gut bacteria, therefore leading to the induction of dysbiosis in the bees’ gut.

## 1. Introduction

Thai commercial honeybee, *Apis mellifera* Linnaeus, 1758, is a key pollinator of many economically important crops. However, the number of honeybees is on the decline due to factors such as ecological changes, pesticide use, bee pathogens [1], and a decrease in the gene pool [2]. In particular, bee pathogens and pesticides directly affect the honeybees’ health from consumption and consequently impact beneficial bacterial symbiosis in the intestine [3]. Bee pathogens such as *Nosema ceranae* (cause of Nosema disease) and *Paenibacillus larvae* (cause of American foulbrood (AFB) disease) propagate in the gut of honeybees [4,5]. Nosema, a microsporidian fungus, infects the epithelial layer of the midgut of adult honeybees, causing digestive disorders and shortening honeybees’ life spans [6]. On the other hand, a spore-forming bacterium *Paenibacillus larvae* infects only honeybee larvae’s midguts [7]. Spores germinating in the midgut proliferate rapidly for several days without destroying the integrity of the midgut epithelium. Shortly afterward, infected individuals turn brown and their bodies becomes a hard scale of material deposited on the side of cells [8]. However, despite the prevalence in America and some parts of continental Europe, *P. larvae* infection has not yet been reported in Thailand [9].

Honeybees live in a symbiotic relationship with many beneficial gut bacteria where the insects provide a habitat for the bacteria. In turn, the bacteria assist honeybees in various physiological roles including nutrient absorption, immunity enhancement, and inhibition of some insect pathogens [10]. Five dominant gut bacteria species, including *Snodgrassella alvi*, *Gilliamella apicola*, two species of *Lactobacillus*, and *Bifidobacterium*, are in a symbiotic relationship with honeybees [11]. The others that could be present in the guts of many honeybees are *Bartonella apis, Apibacter adventoris, Frischella perrara,* and Acetobacteraceae [11]. The roles of core gut bacteria are associated with nutrition as *G. apicola*, *Lactobacillus*, and *Bifidobacterium* can absorb and metabolize a wide range of plant-produced carbohydrates [12]. Moreover, some main gut bacteria have several genes that are involved in carbohydrate metabolism, as evident by genomic studies [13,14,15], and various strains have different carbohydrate metabolizing abilities [13,16,17]. Other bacteria such as *Bartonella apis*, *Apibacter adventoris*, *Frischella perrara*, and Acetobacteraceae are minor bacteria in honeybees, and their functions are still unclear [18].

A study investigating the gut bacteria of honeybees at different life stages and sites [19,20] suggested that the dynamic of bacterial communities has a strong impact on honeybees’ health and sensitivity to ecological changes [20]. However, information on changes in the bacterial community upon the invasion of pathogens and consequent impact on honeybees’ health is still limited.

The advent of next-generation sequencing allows scientists to study bacterial communities in finer and descriptive details because of its efficiency. Moreover, the tool can be utilized to track the dynamic changes of gut bacteria upon pathogen invasion. This study aimed to investigate the impact of *N. ceranae* and *P. larvae* on bacterial communities in the guts of Thai commercial honeybees, *A. mellifera*, using next-generation sequencing. The results from this study would be useful for disease diagnosis and would help to maintain honeybees’ health in apicultural fields.

## 2. Materials and Methods

### 2.1. Sample Collection

Honeybees (*A. mellifera*) were bought from the northern region of Thailand, including Nan, Chiang Rai, and Lumphun (3 hives/province), and were cultured in an experimental site of the Bee Protection (BeeP) center at the Faculty of Science, Chiang Mai University (18°48′13.7″ N, 98°57′22.9″ E).

### 2.2. Sterilized Honeybee Hive Diagnosis

The colonies showed no visible clinical symptoms of disease. Before the experiments, fifty adult bees were randomly collected from each colony, checked for Nosema spores using light microscopy, as described in Cantwell (1970) [21], and confirmed by PCR analysis with the target on locus-SSU-rRNA-genes and their condition described by Chen and colleagues (2008) [22].

Ten 5th instar larvae were randomly selected from each hive with a small grafting tool and were placed onto a petri dish. After that, the last tergum segment of the bee was grabbed and pulled with forceps to obtain the intestine. The intestine was separated, grounded, and resuspended in phosphate-buffered saline (PBS) in a microcentrifuge tube. The suspension was examined under a microscope to look for spores of *N. ceranae*. *P. larvae* infection was not examined, because it is not currently found in Thailand.

### 2.3. Honeybee Preparation for P. larvae Infection

To obtain *A. mellifera* first instar worker larvae, the queen was allowed to lay eggs over the empty brood cells with a queen excluder cage, which allowed only worker bees to tend the queen and prevented drones from traversing to the queen, and the cage was placed into a hive. After 24 h, the queen started to oviposit depending on the cleanliness of the comb and worker bees. The queen was released from the excluder cage after laying eggs (approximately 24–72 h) [23,24]. Afterward, the cage was placed in the middle of the hive so the queen and the larvae could easily be taken care of by nurse bees [23,25]. Four days after the queen had been caged, newly hatched larvae were visible. These larvae were collected with a small grafting tool and placed onto a petri dish for further experiments to identify *P. larvae* [26].

#### 2.3.1. *P. larvae* Inoculum

The *P. larvae* LMG9820 was purchased from the Belgian Co-ordinated Collections of Micro-organisms (BCCM) collection.

T-HCL-YGP (a modification of a TMYGP medium developed by Dingman and Stahly) agar containing (per liter) 15 g of yeast extracted (Difco, Becton Dickinson, MD, USA), 200 mL of 0.1 M of Tris-HCl, 1 g of pyruvic acid (Sigma, St. Louis, MO, USA), 40 mL of 10% glucose with 3 µg/mL of nalidixic acid, pH 7.0, and 20 g of agar was used for bacteria isolation. *P. larvae* was incubated at 37 °C for 7–10 days. The bacteria were kept in a stock of glycerol and stored at −20 °C. A spore suspension was prepared after cultivation for 7–9 days by suspending one full loop of *P. larvae* in a sterile microcentrifuge tube containing 1 mL of sterile distilled water. The suspension was mixed, centrifuged at 18,000× *g* for 10 min, and washed twice with sterile distilled water. The supernatant was discarded, and the pellet was resuspended in 200 μL of sterile water. To reduce contamination and vegetative cells, the suspension was placed in a water bath for 15 min at 80 °C [27]. Endospores were manually counted using a hemocytometer. The suspension was diluted to the concentration of 1 × 10^8^ spores/mL and kept as a stock spore inoculant for further experiments.

#### 2.3.2. Honeybees’ Food Preparation

An artificial diet composition adapted from Rembold et al. (1974) and Fourrier et al. (2015) was used to feed honeybee larvae [26,28] with modifications within the nutrient’s ratios to obtain a diet with 30% of total solids, as recommended by Vandenberg and Shimanuki, (1987) [25]. Briefly, a sugar solution containing 50% sugar and 1% yeast extract (*w*/*w*) in sterile water was filtered through a MF-Millipore™ membrane filter (0.22 μm) (Merck KGaA, Darmstadt, Germany), and royal jelly 50% (*w*/*w*) was added. The diet was prepared for honeybee larvae, stored at 4 °C, and used within 5 days. Adult bees were fed with a solution of sucrose (50% *w*/*w* in water) ad libitum.

#### 2.3.3. *P. larvae* Infection in Honeybee

First instar larvae of *A. mellifera* (*n* = 30) were collected from *P. larvae*-free hives. The larvae were placed in 24-well plates and fed with food supplement mixed with 10^5^ *P. larvae* spores/diet in each individual larva. Six larvae were fed with the diet without *P. larvae* spores and were used as negative controls. The diet was changed daily. Six larvae fed with *P. larvae* spores were collected every day until day 4 (day 0–4). All samples were surface-sterilized. The individual larval gut was kept at −80 °C until the analysis.

#### 2.3.4. *P. larvae* Infection Analysis

Homogenized honeybee samples, larva or adult intestine, were ground using clean pestles in a 1.5 mL microcentrifuge tube with liquid nitrogen. The total RNA was extracted from individual larvae samples using TRIZOL^®^ (Invitrogen, Carlsbad, CA, USA), according to the manufacturer’s recommendations. The RNA quantity was determined by a Micro-Spectrophotometer NANO-200 (Allsheng, Hangzhou Allsheng Inc., Zhejiang, China) and the ratio of 260/280 nm absorbance was used to assess the RNA purity. cDNA synthesis was performed using 300 ng of total RNA and the Tetro cDNA synthesis kit (Bioline, Alexandria, NSW, Australia), as described by the manufacturer. The cDNA was used to perform qPCR with each pathogen-specific primer for each day of pathogen infection. The *P. larvae* PCR primers were ERIC1R (5′-ATGTAAGCTCCTGGGGATTCAC-3′) and ERIC2 (5′-AAGTAAGTGACTGGGGTGAGCG-3′) [29] for the ERIC (erythropoietin-induced) gene. Relative expression analysis was performed using beta actin (F-primer: 5′-TTGTATGCCAACACTGTCCTTT-3′ and R-primer: 5′-TGGCGCGATG ATCTTAATTT-3′) and RpS5 (F-primer: 5′-AATTATTTGGTCGCTGGAATTG-3′ and R-primer: 5′-TAACGTCCAGCAGAATGTGGTA-3′) genes as references and the 2^−∆∆CT^ method [30].

### 2.4. Honeybee Preparation for Nosema infection

Newly enclosed bees (*n* = 60), that were moved (via nurse bees) for one hour, were randomly collected from Nosema-free hives and then were placed in three experimental cages (20 bees/cage). They were kept under a controlled temperature (34 ± 1 °C) and 60–70% humidity [29]. Bees were fed with a solution of sucrose (50% *w*/*w* in water) ad libitum.

#### 2.4.1. Honeybee Pathogen Preparation for *N. ceranae* Inoculum

*N. ceranae* spores from *A. ceranae* at the BeeP center apiary were isolated. Briefly, the infected midguts of honeybees were crushed and removed in distilled water, and they were then filtered through a cotton and centrifuged at 5000× *g* for 10 min. The re-dissolved and purified pellet was used in isolation [31]. The purity of the isolates was confirmed by PCR analysis [22]. The number of spores was counted using light microscopy, as described in Cantwell (1970) [21]. Inoculums were freshly prepared on the day of inoculation and diluted with 50% sucrose solution to obtain the concentration of 10^7^ spores/mL.

#### 2.4.2. *N. ceranae* Infection in Honeybee

Five days after emerging, bees were fed with sugar supplement mixed with 10^5^ *N. ceranae* spores/diet, and another six bees were fed with a diet without spores and were used as negative controls. The food was changed daily. Adult bees were fed with a solution of sucrose (50% *w*/*w* in water) ad libitum. Six honeybees fed with *N. ceranae* spores were collected every day until day 9 (day 0–9). All samples were surface-sterilized. The individual intestine was removed from the bee and kept at −80 °C until the analysis. The bees were examined for the presence of Nosema with pathogen-specific primers for each day of pathogen infection, F-primer (5′-CGGATAAAAGAGTCCGTTACC-3′) and R-primer (5′-TGAGCAGGGTTCTAGGGAT-3′) for *N. ceranae* [22].

### 2.5. Bacterial Community Analysis Using Next-Generation Sequencing

#### 2.5.1. Communities Proportion Analysis

Each honeybee and larval intestine were moved to a 1.5 mL centrifuge tube. The sample was homogenized, and genomic DNA was extracted with the DNeasy Blood & Tissue Kit (Qiagen, Germany). The concentration of the extracted DNA was quantified by the Micro-Spectrophotometer NANO-200 (Allsheng, Hangzhou Allsheng Inc., Zhejiang, China). The genomic DNA was stored at −20 °C until use. The sequencing of 16S rDNA was performed using the 454 pyrosequencing (Roches) platform with V6-V8 region primer pair F: 5′-AAACTYAAAKGAATTGACGG-3′ and R: 5′-ACGGGCGGTGTGTRC-3′. The bacterial 16S rDNA sequences were processed by the Mothur software [32]. The reads were examined for quality and the reads with quality scores more than 30 were analyzed. The SILVA database was used for alignment and removal of chimeric sequences, and then the nonbacterial sequences based on Ribosomal Database Project (RDP) database. Sequences with 97% identities were selected for OTU clustering (SRA accession: PRJNA604681). The output (OTUs microbiome table) was used for the diversity analysis, functional genes prediction, and statistical analysis.

#### 2.5.2. Alpha Diversity and Beta Diversity Analysis

Alpha diversity was performed by PAST software version 3.14 [33] with the Shannon index. For the beta diversity analysis, the NMDS was performed by PAST 3.14, and significance tests of NMDS were analyzed by one-way PERMANOVA with the Bray–Curtis similarity index.

#### 2.5.3. Functional Genes Prediction

Functional gene prediction was performed by the phylogenetic investigation of communities by reconstructing unobserved states (PICRUSt) 1.0.0 (http://picrust.github.io/picrust, accessed on 8 September 2020) based on the MetaCyc database [34]. The pairwise Wilcoxon test (*p* < 0.05) and Kruskal–Wallis test were used for difference analysis of each gene expression.

### 2.6. Statistical Analysis

The significant differences in the levels of pathogenic infection were analyzed by one-way ANOVA using SPSS Statistics version 17 (SPSS Inc. Released 2008. SPSS Statistics for Windows, Version 17.0. Chicago, IL, USA: SPSS Inc.). The beta-diversity and significance between communities were analyzed using Paleontological statistics software PAST 3.14 [33].

## 3. Results

### 3.1. Level of P. larvae Infections and Their Bacterial Diversity Analysis

**Figure 1:**
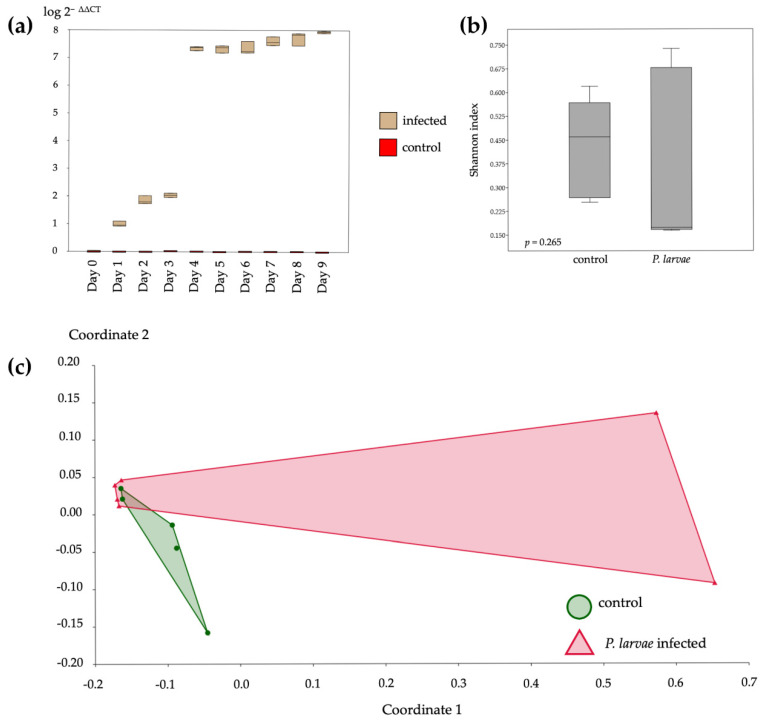
*P. larvae* infection level and diversity analysis of bacterial communities in honey bee larvae. The expression level, calculated by 2^−∆∆CT^ method, of *P. larvae* expression in larvae from day 0 to 9, showing that the *P. larvae* infection was significantly increased from day 3 to 4 and then slightly increased until day 9 (**a**). Alpha diversity, indexed with the Shannon parameter, of bacterial communities of control and infected bees that were not significantly different (*t* = 1.1817, *p* = 0.265) (**b**). The nonmetric multidimensional scaling (NMDS) analysis of bacterial communities between the control and infected groups of honeybees showed that the bacterial communities of *P. larvae*-infected were not significantly different from the control (F = 2.367, *p* = 0.2447), with a stress value of 0.0127 (**c**).

*P. larvae* infection levels increased over the incubation period. The highest *P. larvae* expression level was on day 4 of infection (Figure 1a). The larvae started to die after day 4 and, therefore, larvae on day 4 were selected for the bacterial community investigation. In conclusion, the bacterial communities were analyzed on samples from day 8 for Nosema infection and day 4 for *P. larvae* infection. The bacterial communities’ alpha diversity indicated by the Shannon parameter was not significantly different after the infection by *P. larvae* (*t* = 1.1817, *p* = 0.265) (Figure 1b), and the beta diversity performed by the nonmetric multidimensional scaling (NMDS) method showed that the areas overlapped, which means that the bacterial communities between the control and the infected groups were not significantly different (F = 2.367, *p* = 0.2447), with stress value = 0.0127 (Figure 1c).

### 3.2. Bacterial Communities in the Gut of A. mellifera Infected by P. larvae

The taxonomic classification of bacterial classes in the *A. mellifera* larval gut fed without spores of *P. larvae* (control) showed that the sequences belonged to Alphaproteobacteria (97.80 ± 1.80%), Actinobacteria (0.89 ± 1.21%), Bacilli (0.66 ± 0.43%), and Gammaproteobacteria (0.37 ± 0.21%) (Figure S1). In contrast, *A. mellifera* larvae fed with *P. larvae* showed the presence of Alphaproteobacteria (79.97 ± 4.30%), Actinobacteria (0.98 ± 1.39%), Bacilli (9.28 ± 1.52%), and Gammaproteobacteria (8.73 ± 1.36%) in the gut (Figure S2) with different class level proportions for Bacilli (*t* = 13.39, *p* < 0.001), Alphaproteobacteria (*t* = 9.366, *p* < 0.001), and Gammaproteobacteria (*t* = 11.97, *p* < 0.001).

**Figure 2:**
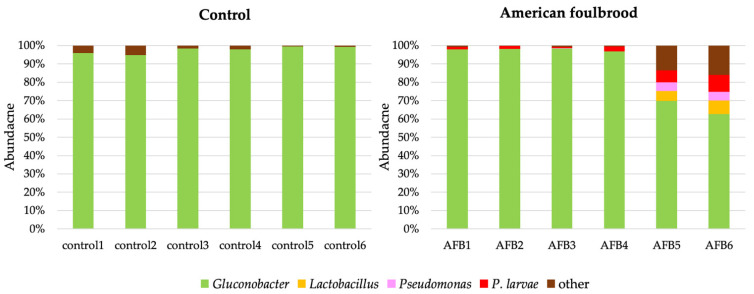
Proportion of bacteria, at the genus level, found in the control and American foulbrood-diseased *A. mellifera* after the bees were infected by *P. larvae*.

At the genus level, the bacteria detected in the control were *Gluconobacter* (97.64 ± 1.85%) and others (2.3 ± 1.85%) (Figure 2). The bacteria detected in the gut of *A. mellifera* larvae fed with *P. larvae* were *Gluconabacter* (90.18 ± 14.36%), *Lactobacillus* (2.32 ± 3.64%), *Pseudomonas* (1.76 ± 2.71%), *Paenibacillus* (3.42 ± 2.17%), and others (2.31 ± 5.03%) (Figure 2). There was an increase in the proportion of bacterial communities of *Lactobacillus, Pseudomonas*, and *P. larvae* in the infected group. All sequences were submitted to BioProject (BioProject ID: PRJNA604681).

### 3.3. Level of Nosema Infections and Their Bacterial Diversity Analysis

**Figure 3:**
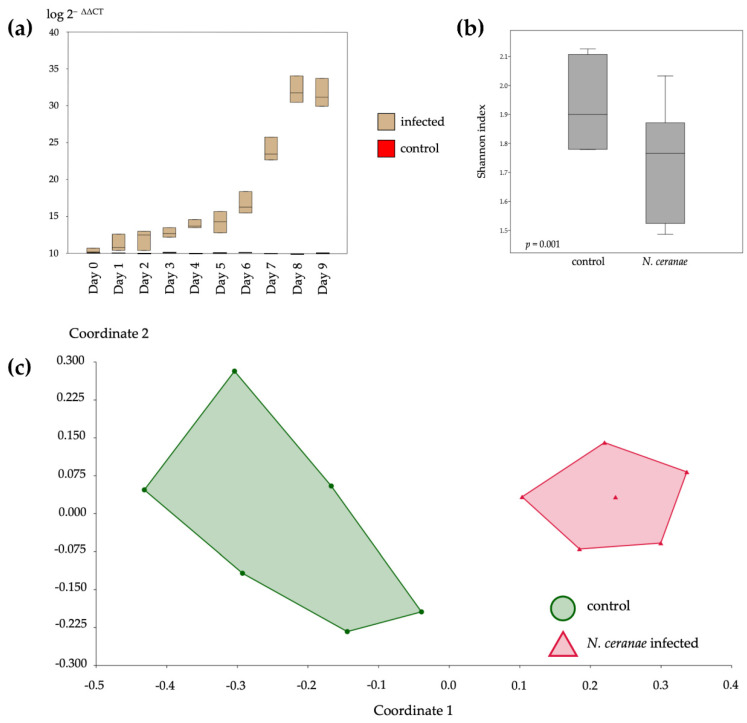
*Nonsema ceranae* infection level and diversity analysis of bacterial communities in honey bee. The expression level, calculated by 2^−∆∆CT^ method, of *N. ceranae* expression in honeybee from day 0 to 9, showing that the *Nosema* infection was increased over the period of 8 days and stabilized on day 9 (**a**). Alpha diversity, indexed with the Shannon parameter, of bacterial communities of control and infected bees that were significantly different (*t* = 4.3832, *p* = 0.001) (**b**). The nonmetric multidimensional scaling (NMDS) analysis of bacterial communities between the control and infected groups of honeybees showed that the bacterial communities of *Nosema*-infected were significantly different from those of the control (F = 10.28, *p* = 0.002), with a stress value of 0.1131 (**c**).

The infection severity of *N. ceranae* increased from day 0 to 8 and then became stable on day 9 (Figure 3a). The highest expression level of *N. ceranae* was observed on day 8. The bacterial communities’ alpha diversity indicated by the Shannon parameter showed a significant difference in bacterial communities in *N. ceranae*-infected honeybees (*t* = 4.3832, *p* = 0.001) (Figure 3b). The beta diversity showed that the bacterial communities infected by *N. ceranae* differed from the control (F = 10.28, *p* = 0.002), with stress value = 0.1131 (Figure 3c).

### 3.4. Bacterial Communities in the Gut of A. mellifera Infected by N. ceranae

The taxonomic classification of bacterial classes in the gut of *A. mellifera* suggested that the majority of sequences from *A. mellifera* fed without spores of *N. ceranae* (control) were of Gammaproteobacteria (34.52 ± 25.49%), Betaproteobacteria (25.75 ± 10.40%), Alphaproteobacteria (22.75 ± 13.96%), and Bacilli (16.58 ± 9.75%) (Figure S2). In contrast, bacteria in the gut of *A. mellifera* fed with *N. ceranae* were Gammaproteobacteria (15.74 ± 16.89%), Betaproteobacteria (40.21 ± 0.12%), Alphaproteobacteria (26.77 ± 0.09%), and Bacilli (16.91 ± 0.04%) (Figure S1). At the class level, we found no significant differences in the proportion between the infected and control group for all.

**Figure 4:**
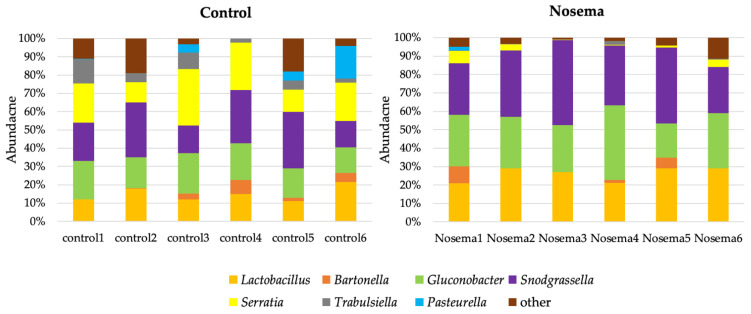
Proportion of bacteria, at the genus level, found in the gut of the control and Nosema-infected *A. mellifera* that showed significant difference, *Lactobacillus* (*t* = 4.75, *p* < 0.001), *Gluconobacter* (*t* = 3.2, *p* = 0.009), *Snodgrassella* (*t* = 2.56, *p* = 0.028), *Serratia* (*t* = 5.33, *p* < 0.001), and *Trabulsiella* (*t* = 3.127, *p* = 0.01).

At the genus level, bacteria detected in the control group were *Lactobacillus* (14.97 ± 4.01%), *Bartonella* (2.92 ± 2.9%), *Glucanobacter* (18.35 ± 3.12%), *Snodgrassella* (23.43 ± 7.58%), *Serratia* (20.42 ± 7.76%), *Trabulsiella* (6.15 ± 4.34%), and *Pasteurella* (4.63 ± 6.94%) (Figure 4). In the *A. mellifera* gut fed with *N. ceranae, Lactobacillus* (26.00 ± 3.95%), *Bartonella* (2.84 ± 12.66%), *Glucanobacter* (28.42 ± 14.41%), *Snodgrassella* (34.86 ± 7.88%), *Serratia* (2.68 ± 17.07%), *Trabulsiella* (0.51 ± 2.08%), and *Pasteurella* (0.33 ± 0.75%) were found (Figure 4). The proportions of bacterial communities between the infected and control groups were significantly different for *Lactobacillus* (*t* = 4.75, *p* < 0.001), *Gluconobacter* (*t* = 3.2, *p* = 0.009), *Snodgrassella* (*t* = 2.56, *p* = 0.028), *Serratia* (*t* = 5.33, *p* < 0.001), and *Trabulsiella* (*t* = 3.127, *p* = 0.01).

### 3.5. Functional Gene Prediction

**Figure 5:**
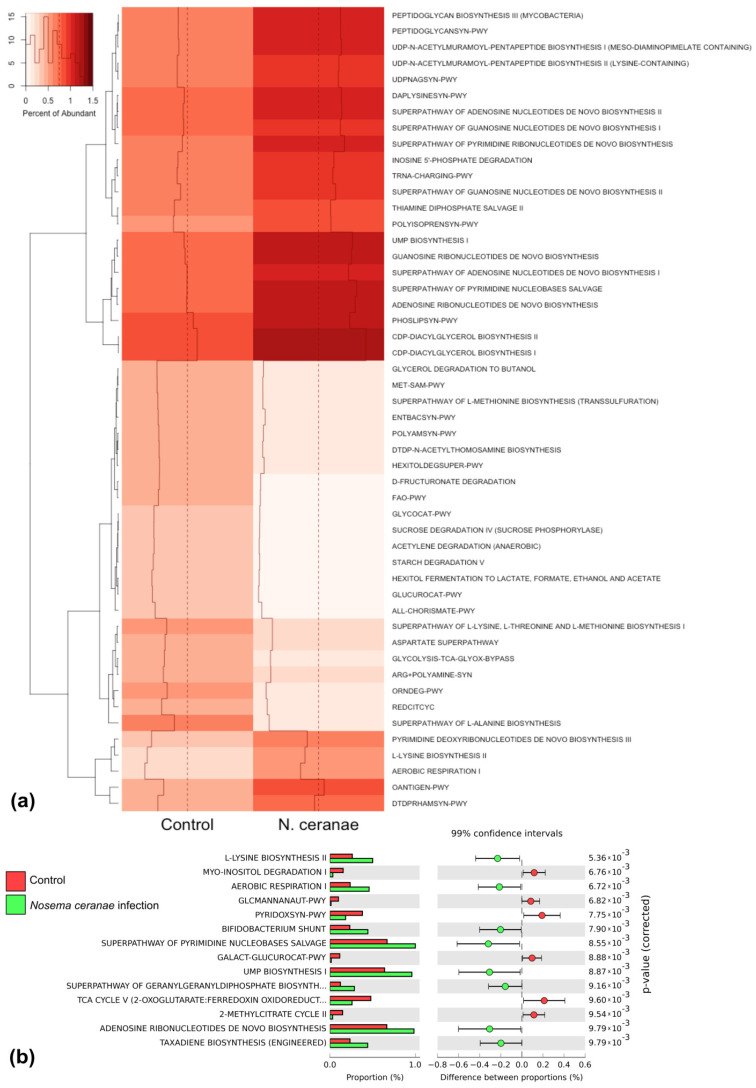
The functional genes prediction analysis of Nosema-infection honey bee. Functional gene expression heat map of control and Nosema-infected *A. mellifera* that showed the different of functional gene (**a**) with part of *p*-value of the difference between proportion chart (**b**). The full *p*-value chart is shown in Figure S4.

The functional genes in *A. mellifera* identified by PICRUSt 1.0.0 were divided into two groups: increasing and decreasing functional activity. The bacterial genes that increased in functional activity after *N. ceranae* infection were genes involved in L-lysine biosynthesis II, aerobic respiration I, Bifidobacterium shunt, superpathway of pyrimidine nucleobases salvage, UMP biosynthesis I, superpathway of geranylgeranyl diphosphate biosynthesis, adenosine ribonucleotide de novo biosynthesis, and taxadine biosynthesis (Figure 5). In contrast, the functional genes of *P. larvae*-infected larvae were not significantly different from those of the control (Figure S3).

## 4. Discussion

We investigated the impact of gut-propagating pathogens, including *N. ceranae* and *P. larvae*, on bacterial communities in the gut of Thai commercial honeybee, *A. mellifera,* by next-generation sequencing. The bacterial community investigation was performed on bees on days where the pathogenic infections were highest, as determined by qPCR.

The infection level of Nosema that increased from day 0 to 8 indicated that Nosema spores were dividing and then became stable on day 9. We considered day 8 of Nosema infection to be the initial day with the highest infection level; therefore, the larvae from day 4 were collected to investigate the microbial community. For *P. larvae* infection, the expression level on day 4 significantly increased from day 3 and, therefore, larvae on day 4 were considered to be the initial day with the highest infection level. In addition, the infected larvae started dying on day 5 after *P. larvae* infection. The *P. larvae* strain ERIC I that was used in this research caused the larvae to enter the mortality stage on day 5 and killed all larvae on day 10–12 post-infection [5,35,36]. A study has shown that on day 4 where we observed the highest infection rate of *P. larvae*, the larvae generated the highest level of phenoloxidase activity to cope with the pathogen [37].

For the *N. ceranae* infection, the percentage of Alphaproteobacteria, Betaproteobacteria and Gammaproteobacteria, and Firmicutes (Bacilli) in adult specimens infected with *N. ceranae* was significantly different from that of the control. At the genus level, infection of *N. ceranae* showed an increase in *Snodgrassella*, core bacteria in honey bees, and *Bartonella*. Studies have shown that colonization by *Snodgrassella* in the adult bee gut promoted immunological responses, such as vitellogenin, the important developmental gene, which was suppressed after being disrupted during early adult life [38,39], and *Snodgrassella* could protect against hemolymph infection by *Escherichia coli* through secretion of an antimicrobial peptide [40]. Moreover, the expressions of phenoloxidase, which is involved in the melanization immune response [41], and endochitinase, which is up-regulated after being infected by *N. ceranae* [42], were lower with more *Snodgrasella* than *Snodgrasella*-free bees. *Bartonella* are minor core bacteria in honeybees [11], implying the possibility of a beneficial influence on immune function. However, their functions are still unclear but hypothesized to be involved in promoting disease resistance in honeybees [43]. The percentage of *Trabulsiella*, *Gluconobacter*, and *Lactobacillus*, which were also normal flora in the adult gut of *A. mellifera* [20,44,45], did not significantly differ in the infected adults, while *Serratia* significantly decreased in the infected gut. *Serratia* has been described in many insects and is responsible for diseases in insect production facilities [46,47,48,49,50]. Nevertheless, some *Serratia* were found to be beneficial and assist in digestion by producing digestive enzymes [51,52]. *Lactobacillus* species were found only in adult insects and likely play a central function in carbohydrate catabolism and, thus, in the nutrition of their hosts [15,20]. *Gluconobacter* is a group of acetic acid bacteria that support the oxidation of alcohols or sugars, leading to the production of acetic acid. *Gluconobacter* species are commonly found in soil and associated with plants [53], indicating that the bees gained some bacteria from food in the natural environment. However, *Gluconobacter* was not affected by Nosema infection in honeybees.

*P. larvae* generally infect only bee larvae and, therefore, we studied the effect of *P. larvae* on gut bacteria at the larval stage. The result indicated that the dominant gut bacteria of both control and diseased larvae were Proteobacteria (Alphaproteobacteria and Gammaproteobacteria), Firmicutes, and Actinobacteria. This is consistent with previous studies, suggesting that *P. larvae* did not affect dominant bacteria in larval gut [20]. At the genus level, we found *Gluconacetobacter*, a group of acetic acid bacteria, in the gut of *P. larvae-*infected larvae. A recent report in *Drosophila* indicated that *Gluconacetobacter* was involved in the regulation of the innate immune system [54]. Thus, the reduction in *Gluconacetobacter* may affect the immune system and result in a decreased survival rate of *A. mellifera* larvae. *Pseudomonas* was found only in the gut of *P. larvae*-infected larvae, indicating that some bacteria from the environment invade the larvae after the hosts become weak. A study has shown that *Pseudomonas aeruginosa* was able to inhibit pathogenic fungi, and *Pseudomonas fluorescens* caused lethal infections in insect larvae [55]. Therefore, the bee larvae infected by *P. larvae* could become weak and easily attacked by pathogenic *Pseudomonas*.

The functional genes prediction showed changes in the gene expression in control and Nosema-infected bees. The expression of eight functional genes increased after the infection including:

(1) L-lysine biosynthesis II, which was found mostly in Gram-positive bacilli [56] such as *Lactobacillus* found in the infected larvae. Moreover, this function was found in the *Bartonella, Gluconobacter, Snodgrassella, Serratia,* and *Pasteurella* of infected larvae [57,58,59].

(2) Aerobic respiration I, the function of all bacteria in the bees except for *Lactobacillus* [57,58,59].

(3) Bifidobacterium shunt, which is a unique fructose-6-phosphate phosphoketolase pathway of Bifidobacteria for carbohydrates fermentation [60]. However, Bifidobacterium did not significantly differ in the Nosema-infected larvae, indicating that some bacteria could have the functional gene that was similar in function to *Bifidobacterium*.

(4) Superpathway of pyrimidine nucleobases salvage that salvages pyrimidine nucleobase uracil to generate UMP related to the (5) UMP biosynthesis I pathway [61].

Other pathways, including (6) superpathway of geranylgeranyl diphosphate biosynthesis, (7) adenosine ribonucleotide de novo biosynthesis, and (8) taxadine biosynthesis, were generally bacterial functional activities. The increased activities of these pathways also corresponded with the higher level of infection.

The increase in level of functional genes indicated that the bacteria increased in number or an elevated expression of genes occurred as defense mechanisms against *N. ceranae*. On the other hand, the decreased expression of functional genes indicated gene suppression by Nosema infection, or the decrease in bacteria in the larval gut or in functional activity of the host. Further studies are warranted to investigate the interaction between bees and pathogens and to provide further guidance on pathogen control in the apicultural industry, especially the restoration of lost gut microbiota or bacteriotherapy as in human [62,63] because changing the pathogen strains or bee species could affect the outcome of the study [64].

## 5. Conclusions

*N. ceranae* infection increased the level of *Snodgrassella* and *Bartonella* in the adult bees’ gut, while that of *Serratia* decreased. However, *P. larvae* had no effect on the bacterial community in the larval gut. Moreover, opportunistic *Pseudomonas* was found to occupy the larvae gut after *P. larvae* infection. These results would be useful to strategize disease diagnosis and maintenance of honeybee health in apicultural fields.

## Data Availability

The sequences generated in this study were submitted to NCBI GenBank and are also available from the corresponding author (Terd Disayathanoowat) on reasonable request.

## Supplementary Materials

The following are available online at https://www.mdpi.com/article/10.3390/insects12060525/s1, Figure S1: Proportion of bacteria, at the class level, found in the guts of control and American foulbrood-diseased *A. mellifera*., Figure S2: Proportion of bacteria, at the class level, found in the control and Nosema-infected *A. mellifera*., Figure S3: Functional gene expression heat map of control and *P. larvae*-infected *A. mellifera* larvae. The mostly functional genes of the infected were not different from the control, Figure S4: The *p*-value chart of the difference between proportions of control and Nosema-infected *Apis mellifera* with 99% confidence.
