# Supplementary material for: Impact of Nosema Disease and American Foulbrood on Gut Bacterial Communities of Honeybees Apis mellifera
Source: Insects. 2021 Jun 6;12(6):525. doi: 10.3390/insects12060525 (PMC8227250; doi:10.3390/insects12060525)
Supplement: Supplementary file 1 [file insects-12-00525-s001.zip › Figure S1.pdf]

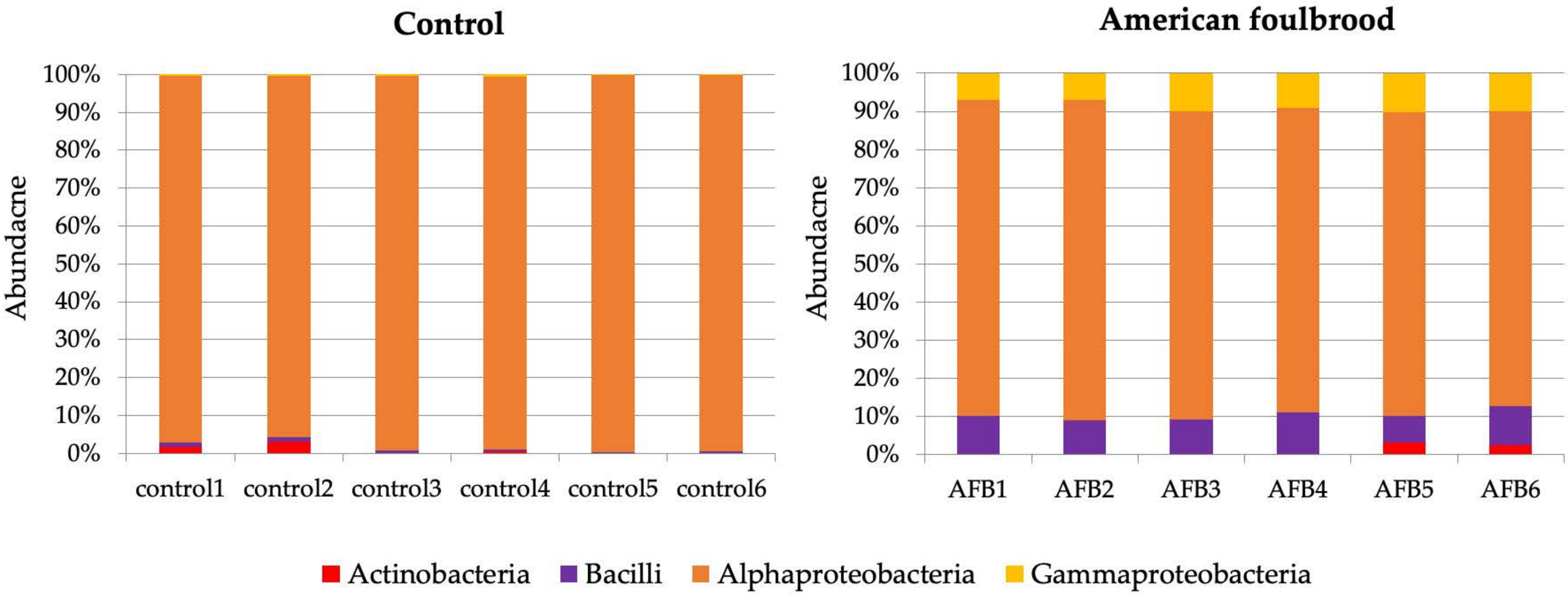

Control
American foulbrood
Abundacne
100%
90%
80%
70%
60%
50%
40%
30%
20%
10%
0%
control1
control2
control3
control4
control5
control6
AFB1
AFB2
AFB3
AFB4
AFB5
AFB6
Actinobacteria
Bacilli
Alphaproteobacteria
Gammaproteobacteria
