# Supplementary figures and images for: Impact of Nosema Disease and American Foulbrood on Gut Bacterial Communities of Honeybees Apis mellifera
Source: Insects. 2021 Jun 6;12(6):525. doi: 10.3390/insects12060525 (PMC8227250; doi:10.3390/insects12060525)

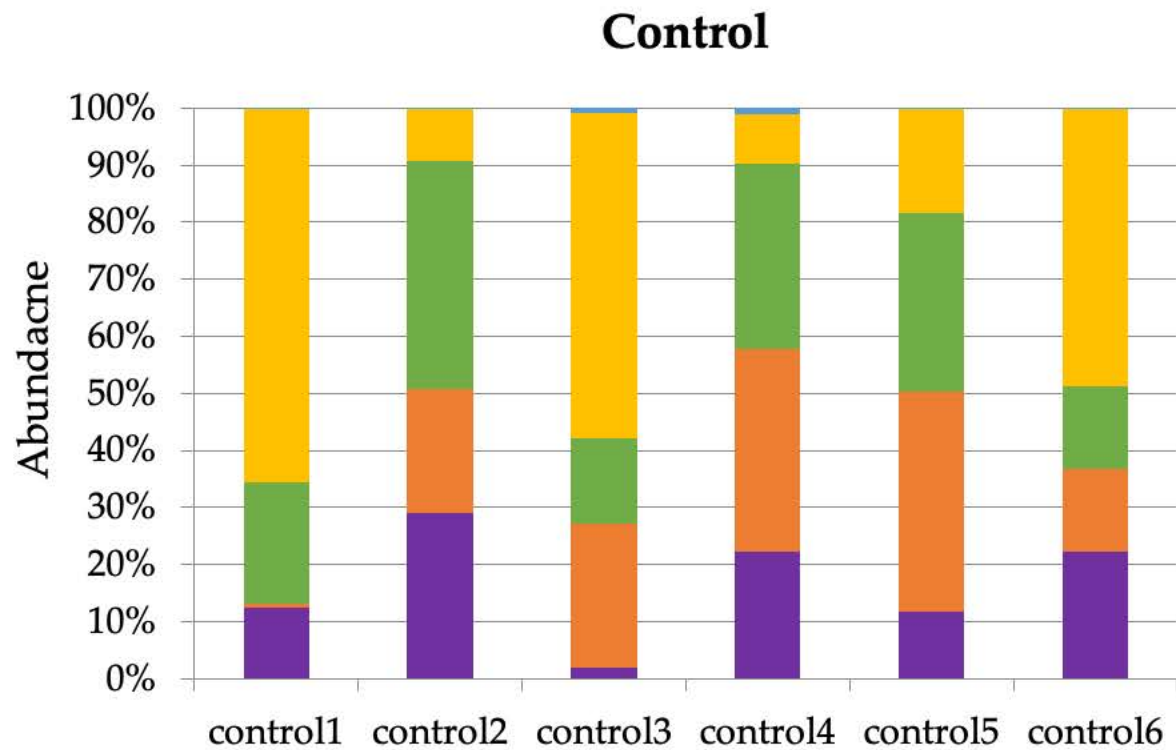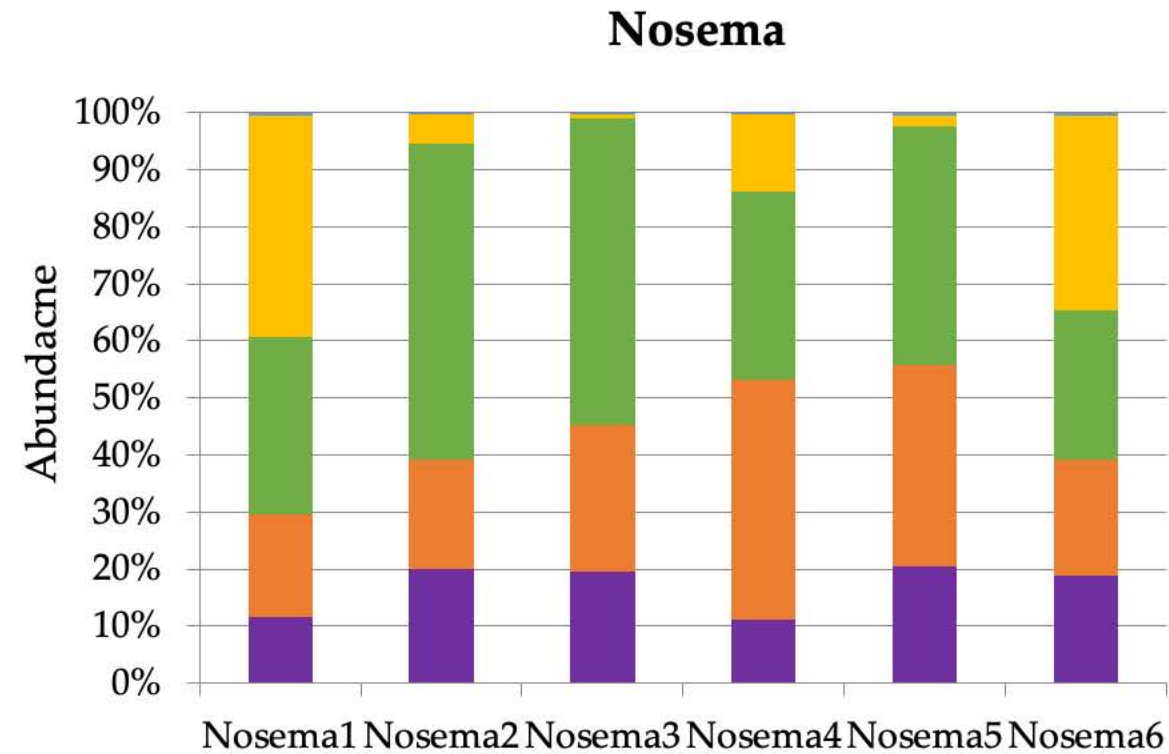

Bacilli

Betaproteobacteria

Other

Alphaproteobacteria

Gammaproteobacteria

Supplement: Supplementary file 1 [file insects-12-00525-s001.zip › Figure S2.pdf]

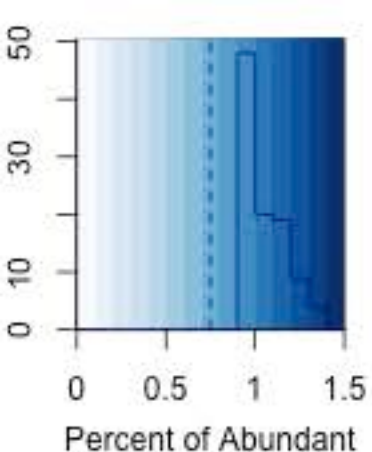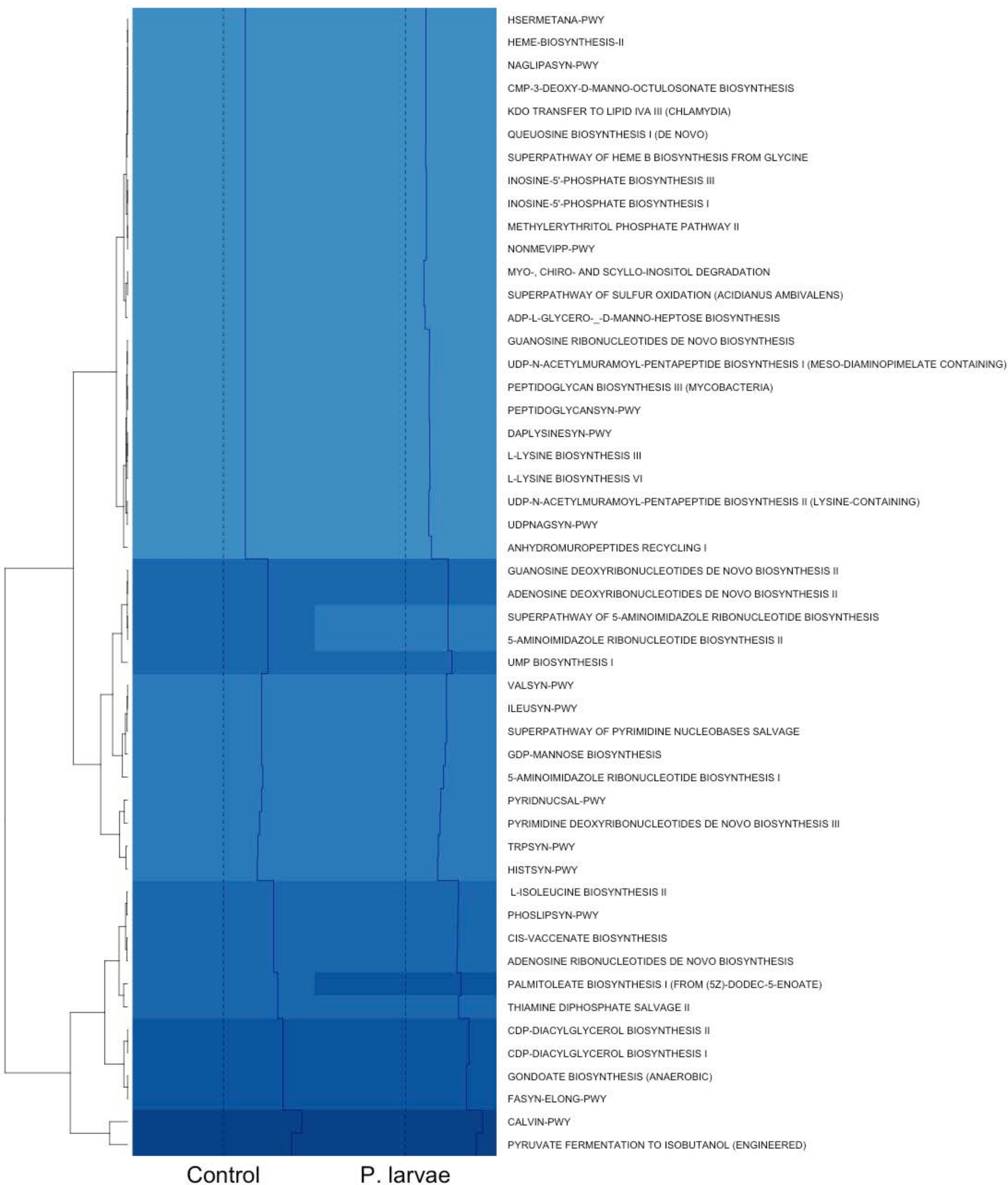

Supplement: Supplementary file 1 [file insects-12-00525-s001.zip › Figure S3.pdf]

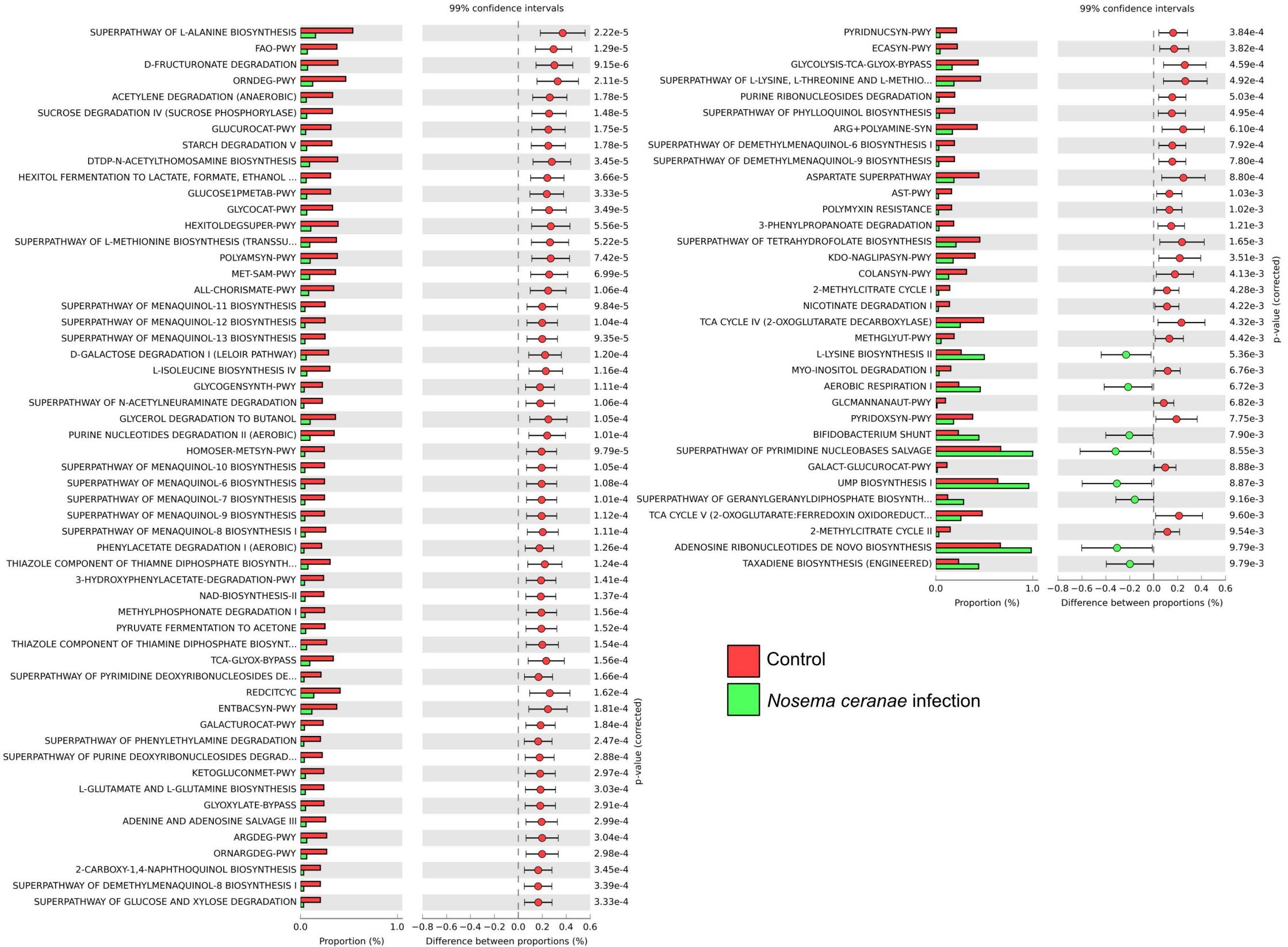

Supplement: Supplementary file 1 [file insects-12-00525-s001.zip › Figure S4.pdf]
